# Cleaner air reveals growing influence of climate on dissolved organic carbon trends in northern headwaters

**DOI:** 10.1088/1748-9326/ac2526

**Published:** 2021-09-21

**Authors:** Heleen A de Wit, John L Stoddard, Donald T Monteith, James E Sample, Kari Austnes, Suzanne Couture, Jens Fölster, Scott N Higgins, Daniel Houle, Jakub Hruška, Pavel Krám, Jiří Kopacek, Andrew M Paterson, Salar Valinia, Herman Van Dam, Jussi Vuorenmaa, Chris D Evans

**Affiliations:** 1 Norwegian Institute for Water Research, N-0349 Oslo, Norway; 2 Centre for Biogeochemistry in the Anthropocene, University of Oslo, N-0315 Oslo, Norway; 3 US EPA, Corvallis, OR 97333, United States of America; 4 UK Centre for Ecology and Hydrology, Lancaster Environment Centre, LA1 4AP Bailrigg, Lancaster, United Kingdom; 5 Environment and Climate Change Canada (ECCC), Montreal, QC H2Y 2E7, Canada; 6 Swedish University of Agricultural Sciences, SE-75007 Uppsala, Sweden; 7 IISD Experimental Lakes Area, Winnipeg, MB R3B 0T5, Canada; 8 Czech Geological Survey, 152 00 Prague, Czech Republic; 9 Global Change Research Institute, CAS, 603 00 Brno, Czech Republic; 10 Biology Centre, CAS, Institute of Hydrobiology, 37005 Ceske Budejovice, Czech Republic; 11 Dorset Environmental Science Centre, Ontario Ministry of the Environment, Conservation and Parks, Dorset, ON, Canada; 12 Swedish Environmental Protection Agency, 106 42 Stockholm, Sweden; 13 Consultancy for Water and Nature, NL-1034 WR, Amsterdam, The Netherlands; 14 Finnish Environment Institute, 00790 Helsinki, Finland; 15 UK Centre for Ecology and Hydrology, LL57 2UW Bangor, United Kingdom

**Keywords:** sulfate deposition, carbon cycle, precipitation, catchment DOC export, surface water browning, organic matter solubility

## Abstract

Surface water browning, the result of increasing concentrations of dissolved organic matter (DOM), has been widespread in northern ecosystems in recent decades. Here, we assess a database of 426 undisturbed headwater lakes and streams in Europe and North America for evidence of trends in DOM between 1990 and 2016. We describe contrasting changes in DOM trends in Europe (decelerating) and North America (accelerating), which are consistent with organic matter solubility responses to declines in sulfate deposition. While earlier trends (1990–2004) were almost entirely related to changes in atmospheric chemistry, climatic and chemical drivers were equally important in explaining recent DOM trends (2002–2016). We estimate that riverine DOM export from northern ecosystems increased by 27% during the study period. Increased summer precipitation strengthened upward dissolved organic carbon trends while warming apparently damped browning. Our results suggest strong but changing influences of air quality and climate on the terrestrial carbon cycle, and on the magnitude of carbon export from land to water.

## 1. Introduction

Surface water browning [1], resulting from increasing concentrations of dissolved organic matter (DOM), has been extensively documented in northern carbon-rich ecosystems. A possible consequence of increased DOM is a shift in the terrestrial carbon balance towards greater export from land to sea, of significance to the global carbon balance [2,3]. Indeed, mounting evidence indicates increases in dissolved organic carbon (DOC) along the entire aquatic continuum from headwaters to coastal seawaters [4, 5], with potential impacts on aquatic life [6–8], transport of pollutants and contamination of aquatic food webs [9], greenhouse gas emissions, and drinking water provision [10, 11].

Browning has been attributed to environmental drivers operating on a regional scale, e.g. reduced atmospheric concentrations of pollution-derived sulfur (S) and chloride (Cl) [1], and consequent reductions in their deposition. The mechanism behind the influence of acidic deposition on DOM was first highlighted by Krug and Frink [12], who proposed that the acidification of soils during periods of high atmospheric deposition (particularly of S) decreased the solubility of DOM, and resulted in lower export of organic acids from soils to surface and groundwaters. Changes in soil water acidity and ionic strength are known to affect the protonation and ion sorption of DOM to soil organic matter; the resulting changes in charge density and hydrophobicity produce higher rates of organic matter (de)sorption or precipitation [13]. Similar mechanisms control organic matter flocculation in mixing zones of river and seawater [14] and removal of micro-algae biomass for renewable energy [15]. Increased DOM solubility in soils recovering from acidic deposition has now been demonstrated in soil solution surveys [16], and laboratory [17] and field experiments [18, 19], and the underlying mechanisms can be simulated with physical chemistry models [20]. Recent in-depth studies of the chemical nature of increasing DOM suggest that newly exported organic C is of recent terrestrial origin, rather than from the destabilization of long-term soil C as might be expected from climate change [21]. Reduced levels of atmospheric deposition result from clean air policies in Europe and North America targeting emissions of acidifying pollutants, especially sulfuric acids [22–24]. Reductions in the amount of sea-salt deposition [25] have also been linked to browning in some coastal regions.

Climate also exerts strong controls of DOM, as evidenced by attribution of seasonal [26] and interannual variation [27] in DOM to climate fluctuations. Recently, evidence that a changing climate could also be influencing variability in DOC has received increasing attention, especially regarding hydrological effects [4,28]. For example, shifts in hydrological routing of runoff associated with higher precipitation and discharge can result in markedly higher export of DOM from soils to surface waters [29], with potential impacts on carbon sink strengths of terrestrial eco-systems [3]. Land-use, especially increases in forest productivity, has also been suggested as a driver of long-term change in DOM [30]. Contrary, however, to the transboundary, and continental to global-scale changes in atmospheric pollution and climate, systematic changes in land use and management tend to arise from policy enacted at national to local levels and cannot, therefore, account for the spatial scale of recent DOM increases.

International clean air policies have resulted in strong reductions in S deposition since 1990 [15, 24], especially in Europe, with smaller reductions in recent years. Consequently, sulfate (SO_4_) concentrations of surface waters declined strongly during the 1990s–2000s, but have begun to level off more recently [31]. Given the strong relationship between trends in SO_4_ and DOC, a comparable deceleration in DOC increase might be expected. At the same time, however, the effects of anthropogenic climate change on physical and biogeochemical processes, may be becoming increasingly important in influencing the direction and strength of DOC concentrations and fluxes. Currently, a regional appraisal of recent trends and possible driving factors in DOM is lacking.

Here, our objective is to test if rates of browning have changed, and whether such changes can be quantitatively related to our hypothesized regional-scale environmental drivers, i.e. atmospheric chemistry and climate. We then apply our findings to estimate how changes in concentrations may have influenced rates of transport of DOM to the oceans. To accomplish this, we extended the database presented in Monteith *et al* [1] in both space and time. Note that the monitoring sites whose data we have compiled occupy the temperate and boreal regions of northern Europe and northern North America that have historically received elevated inputs of acidic deposition. While the destabilization of permafrost is one of the most dramatic predicted outcomes of climate change, the current study does not include any catchments where permafrost is a major component of soils. The dataset comprises records from headwaters draining natural and semi-natural catchments that span wide gradients of productivity and vegetation cover (from subarctic tundra to temperate productive forests and grasslands); climate (mean annual temperature, —0.5 °C to +8.0 °C (95%-percentile ranges for 1990–2016)); mean annual precipitation (530–1940 mm) and atmospheric loading of sulfur (high end: 20 [32] to 40 [33] kg S ha^−1^ yr^−1^ in the early 1990s in Central Europe; low end: <1–2 kg S ha^−1^ yr^−1^ around 2015 in Northern Scandinavia [34]) and seasalt influences [31]. Most sites were originally selected for the monitoring of air pollution effects on water quality only, excluding sites with direct human disturbance such as agriculture and forest management in their catchments. The exception was the UK where some catchments have low-intensity grazing and non-native conifer forest plantations [35].

We determined the strength of DOC trends (ΔDOC) for 1990–2016, 1990–2004 (referred to as ‘early’, corresponding with the period presented in Monteith *et al* [1]) and 2002–2016 (referred to as ‘late’). Each period contains the same 426 sites. Trends in measured water chemical variables (SO_4_ and Cl concentrations, separate and summed), and modeled air temperature (*T*) and precipitation (*P*) (annual and summer averages (*T*) and sums (*P*)) were used as proxies for changes in atmospheric chemistry [1] and climate. Changes in land to sea DOC transport were estimated using DOC concentrations and trends from our database in combination with riverine water transport data.

## 2. Materials and methods

### 2.1. Water chemical monitoring programs

Water chemistry data were compiled for the period 1990–2016 from regional and national monitoring programs in: Canada (114 sites); Czech Republic (7); Finland (26); the Netherlands (3); Norway (83); Poland (4); Slovakia (12); Sweden (83); the United Kingdom (21); and the United States (75). Records represent mostly headwater lakes and lower-order streams from natural or semi-natural catchments, i.e. largely free of local disturbance, other than forestry and rough grazing practices in sites in the UK [35]. Because we use surface water concentrations of SO_4_ and Cl as surrogates for S and Cl deposition, we excluded sites where other processes (e.g. road salt, mine drainage, glacial melting) produce anomalies in those ions. All monitoring programs follow well-established methods and analytical procedures [31, 36]. Sampling frequency ranged from annual to weekly, usually with a higher sampling frequency for streams than for lakes. Previously, temporal patterns in DOC in streams and lakes were found to be similar [37].

### 2.2. Water chemistry

For each monitoring site, we calculated annual median concentrations of DOC, as well as several potential chemical drivers of DOC change (SO_4_, Cl, pH, nitrate [NO_3_], and divalent base cations [ƩCa + Mg]). We used changes in measured surface water concentrations of SO_4_ and Cl to represent change in deposition of S and Cl respectively (from both deposition and seasalts). This was because site-specific deposition estimates were generally unavailable for the monitoring sites, modeled estimates often provide wet deposition estimates only, with considerable uncertainty regarding absolute levels, and no estimates were available for the contribution from seasalts. Non-marine concentrations of SO_4_ and base cations were also calculated [38] and used as potential chemical explanatory variables.

### 2.3. Climate

Gridded daily mean air temperature and precipitation values (0.5° resolution) were downloaded from the Climate Research Unit, University of East Anglia (https://crudata.uea.acuk/cru/data/hrg/; dataset CRU-TS-v3.24.01). Each site was matched to a grid cell (several monitoring sites may be associated with a single grid cell), and daily temperature and precipitation were converted to monthly and annual mean temperature and sum of precipitation. Summer mean temperature and precipitation sum were calculated for July, August and September (JAS).

### 2.4. Trend analysis

We applied the Mann-Kendall test [39] to median chemical and mean climate variables to produce estimates of annual trends for DOC and all possible drivers. The magnitudes of the trends were estimated with the Theil–Sen estimator [40]. Trends were calculated for the periods 1990–2016, 1990–2004 and 2002–2016. The periods were determined on the rationale that (a) robust trend analyses require at least ten years of records, but preferably more, (b) a slight overlap prevents that the transition period plays a disproportionate role in the analysis, (c) 1990–2004 was used in Monteith *et al* [1], thus allowing for a direct comparison with their previously published observations. Sites were included only where trends could be calculated for all three periods of interest, i.e. sites that had (a) data for at least 65% of the total number of years in the period; (b) at least two values in the first four years of the period of interest, (c) at least two values in the last four years of the period of interest. To transform absolute changes in DOC into relative changes in % yr^−1^, we multiplied Theil–Sen slopes by 100 and divided by the site median concentration for the given period.

### 2.5. Statistical modeling

Regression analyses were performed using the slope of DOC change (ΔDOC) as the dependent variable, and a range of chemical and climatic variables as candidate predictors, along with interaction variables combining change and state variables (table 1).

Best subsets modeling was performed using the bestglm package in R [41]. Rather than relying on a stepwise approach to adding candidate variables to a regression model, the best subsets approach tests all possible combinations of the predictor variables, and then produces a list of the «best» model(s) according to some statistical criteria (in this case, Akaike information criterion (AIC), which balances the trade-off between the goodness of fit of the model and the simplicity of the model).

Further data analysis included exploration of the relative contribution of various potential drivers (e.g. chemical and climatic variables) in the three time periods (i.e. the full record (1990–2016); the first 15 years (1990–2004; referred to as «early»); and the last 15 years (2002–2012; referred to as «late»)). We used the realimpo package in R [41] to compute the proportion of *R*^2^ that each predictor variable contributes using the lmg metric [42]. The technique uses variance decomposition to average the improvement in *R*^2^ gained by adding each candidate predictor to multiple linear regression models of various sizes and ‘quantifies the relative contributions of the regressors to the model’s total explanatory value by averaging sequential sums of squares over orderings of regressors’ [43]. The models were run with six variables (the four primary dynamic drivers, plus two interactions between the chemical variables and median (Ca + Mg)), for a total of 720 model iterations (for each time period). The relative importance of each variable is presented as a share of the unexplained variance, and together sum to 100%. For the purpose of our analysis, we included all possible forms of each dynamic driver, and summed their lmg values to represent their overall effect (e.g. for SO_4_, we summed the lmg values for ΔSO_4_ and the interaction of ΔSO_4_ and median (Ca + Mg)).

### 2.6. Calculation of riverine DOC export

We used publicly available estimates of global runoff data (Global Runoff Data Centre; www.bafg.de/GRDC/EN/Home/homepage_node.html; https://geoportal.bafg.de/mapapps/resources/apps/GRDC_FWF/index.html?lang=en) for three European and three North American drainage basins in combination with median DOC concentration values to calculate changes in riverine DOC export to coastal regions. Drainage basins with substantial regional coverage (Baltic Sea, Norwegian Sea, North Sea; Gulf of St Lawrence, North East Shelf, Scotian Shelf) of sites in our database were included, while drainage basins with limited data (Barents Sea and Newfoundland Shelf) were not. Averaged annual runoff data for 1961–2016 were compiled, and runoff was converted to mm yr^−1^ using the reported area for the drainage basins. None of these regional annual runoff time series showed a long-term trend (figure S4 (available online at stacks.iop.org/ERL/16/104009/mmedia)). Sites were attributed to drainage basins based on location. DOC flux at the start of the period of interest (1990–2006) was estimated as the median DOC concentration (across all sites in the basin) for the initial five years (1990–1994), multiplied by the mean annual runoff; these fluxes are assumed to represent flux values in 1992. Estimated changes in DOC export were calculated by applying the median rate of DOC change for all sites in each basin to the estimates of flux in 1992, and then multiplied with time. Thus, the changes in DOC flux we present are driven entirely by the measured changes in DOC concentrations, with export of water assumed to be unchanged during the period of interest.

## 3. Results

### 3.1. Browning of surface waters continues

**Figure 1.**
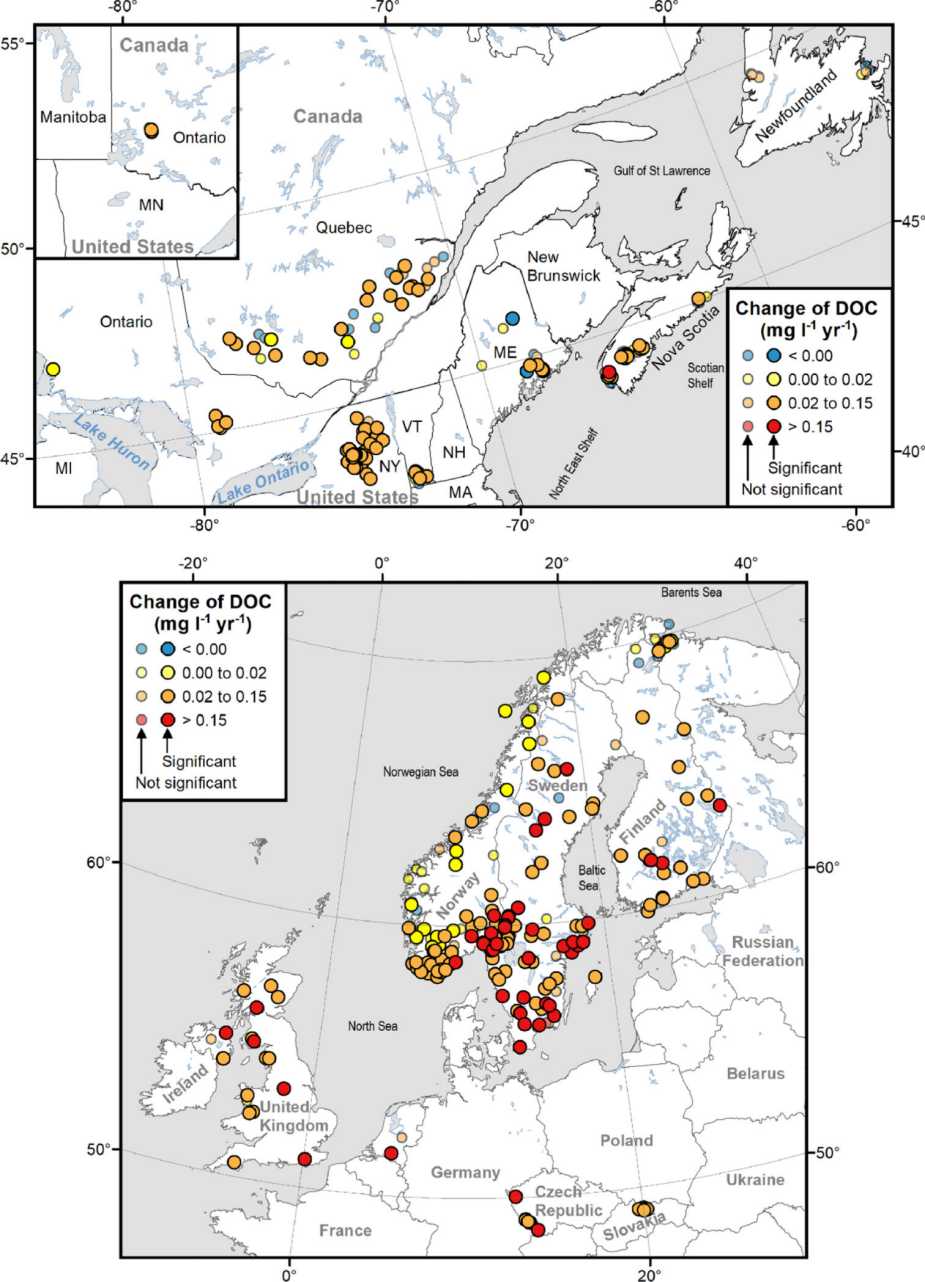
Trends in DOC (mg C l^*−*1^ yr^*−*1^ ) for the period 1990–2016. Upper panel shows sites in North America and lower panel for Europe.

Over the period 1990–2016, ΔDOC was overwhelmingly positive (figure 1; supplementary information (SI), figure S1), with 383 of 426 sites showing positive slopes (269 significant, *p* < 0.05). Only 43 sites showed negative slopes, four of which were significant (*p* < 0.05). As demonstrated elsewhere [1,4], the magnitude of ΔDOC depended strongly on the median DOC concentration (figure S2). Long-term average concentrations of DOC can be considered as surrogates for catchment carbon pools [44, 45], while also being partly dependent on the extent of dilution by precipitation [4, 35, 45].

**Figure 2.**
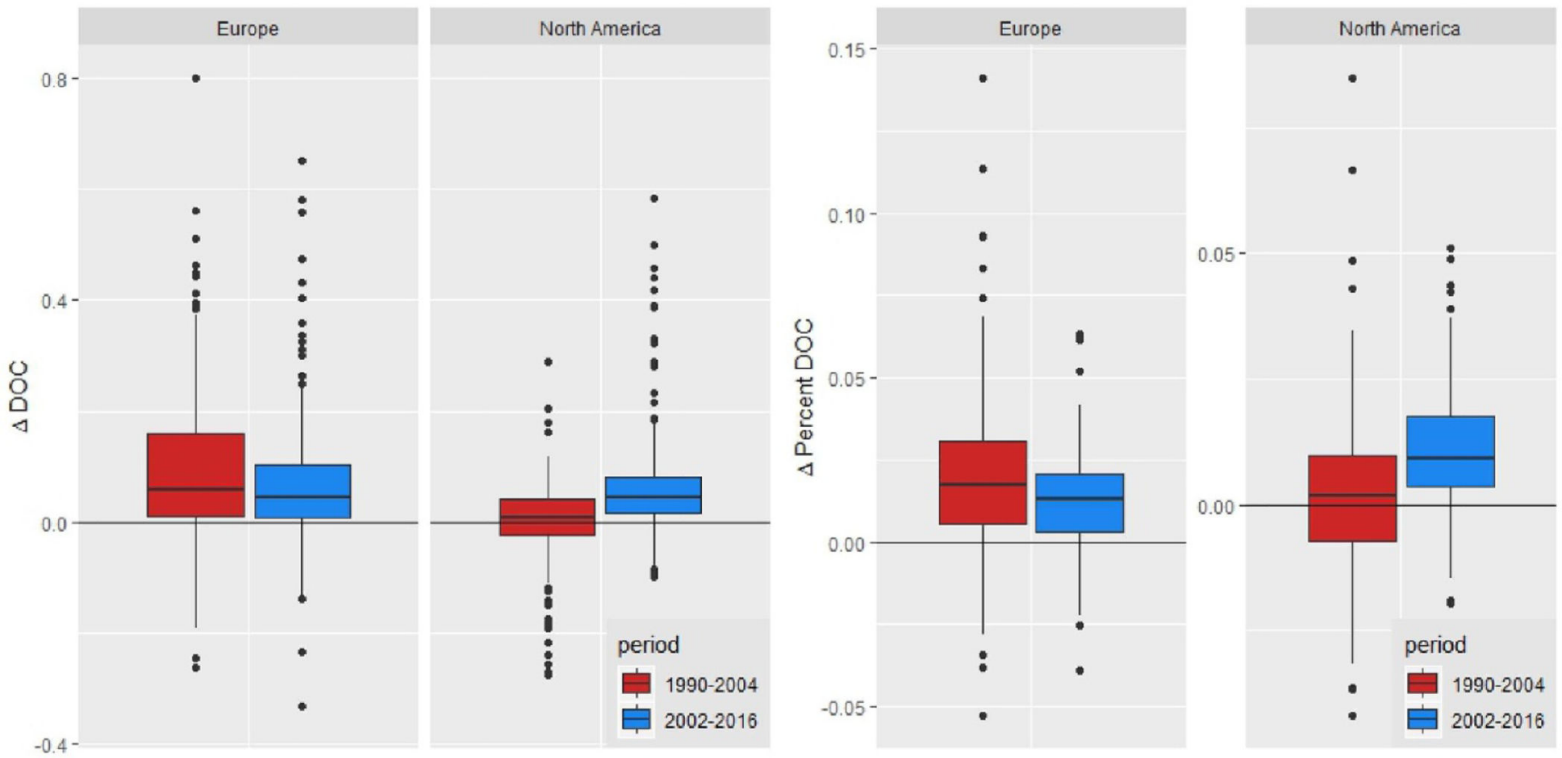
Box and whisker plots of absolute (mg C l^*−*1^ yr^*−*1^ ) (left panel) and relative (% yr^*−*1^ ) (right panel) DOC change, organized by continent and calculated for each of two time periods (1990–2004 and 2002–2016). Note that the *y*-axis for Europe and North America for the relative DOC change are different.

Overall, median ΔDOC was higher in the 2002–2016 period than in 1990–2004, but the median proportional rate of change, expressed as %ΔDOC (100 × ADOC divided by median DOC concentration), was not (table 1). Thus, across the full dataset, rates of browning have continued at a similar pace to earlier [1]. However, browning rates accelerated in North America whereas they slowed in Europe (figure 2). For the entire period, %ΔDOC in Europe was approximately twice as high as in North America (table 1).

### 3.2. Deposition and climate both exert influence on DOC trends

The contrasts in regional patterns and rates of browning call for a further analysis of potential controls. We used a best subsets approach to multiple regression to identify critical drivers of ΔDOC (1990–2016), starting with a comprehensive list of chemical and climatic independent variables and their interactions (table S1). Using the AIC we observed that model fitness plateaued at six or seven variables. The best models that could be achieved using fewer variables (e.g. <4) consisted exclusively of chemical variables.

**Figure 3.**
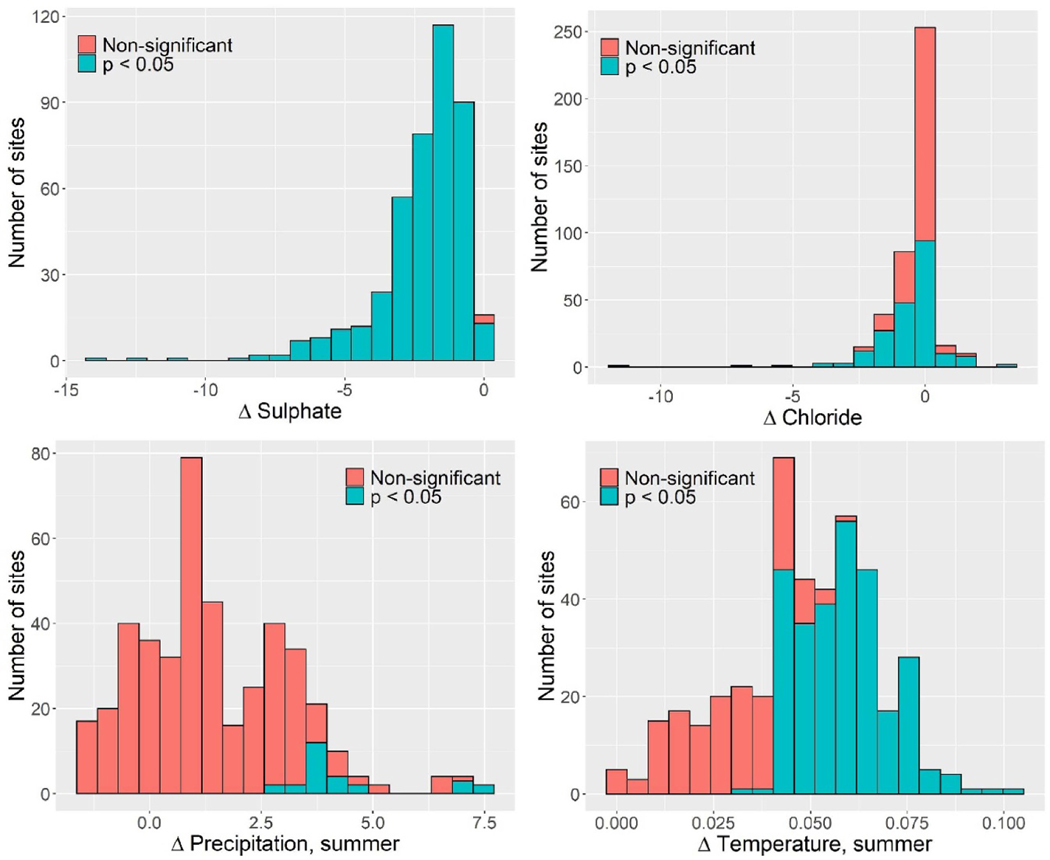
Histogram of trends distribution in key potential drivers of DOC (slopes in *μ*eq l^***−***1^ yr^*−*1^ for sulfate and chloride; slopes in **°**C yr^*−*1^ and mm yr^*−*1^ for temperature and precipitation, respectively) for the period 1990–2016. Summer is defined as July–September.

**Figure 4.**
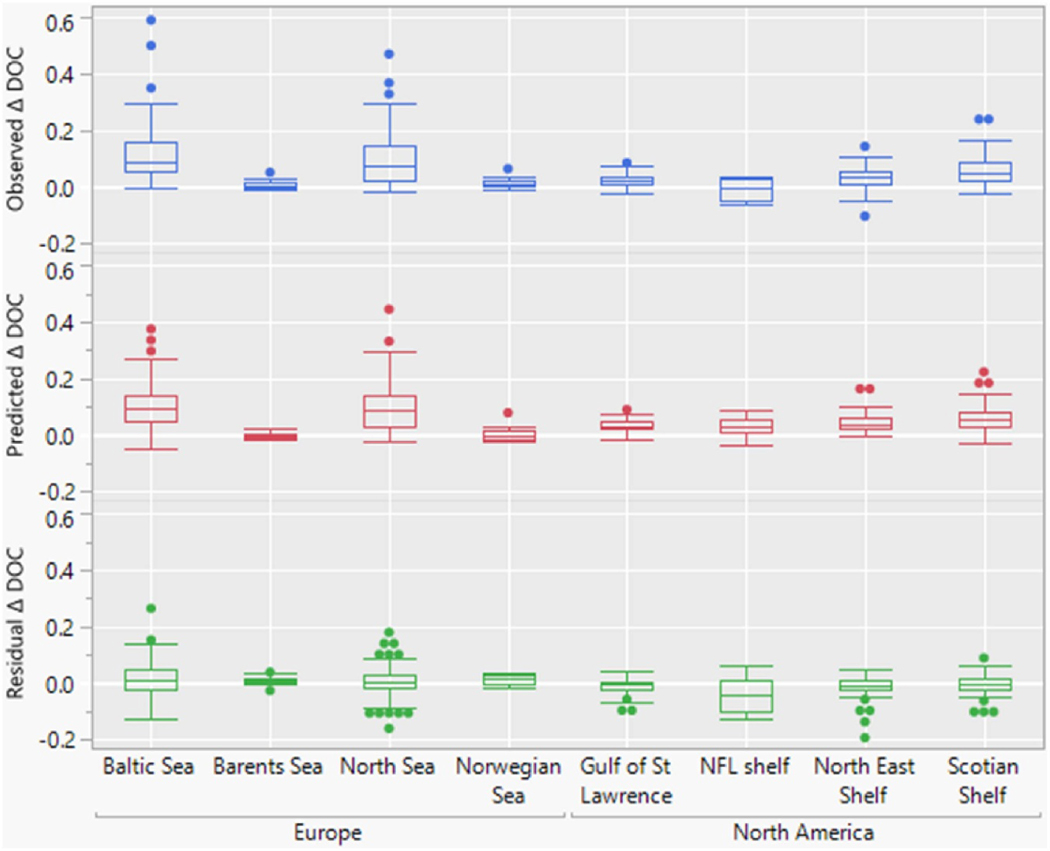
Box and whisker plots of DOC change (1990–2016) expressed as Sen slopes in mg C 1^***−***1^ yr^***−***1^ (top, observations; middle, predicted by best model; bottom, residual trends), presented by drainage area (source: Global Runoff Data Centre) and continent. NFL shelf, Newfoundland Shelf.

The seven-variable model (six predictors and one interaction) with the lowest AIC explained 67% of the variance in ΔDOC (table 2) and emphasized the importance of median DOC and the chemical drivers (ΔSO_4_ and ΔCl) for ΔDOC. The model coefficients imply that declines in SO_4_ and Cl deposition (figure 3) exert positive effects on DOC concentrations, in agreement with earlier findings [1]. The interaction between median divalent base cation concentrations and deposition implies that reduced deposition has a greater effect in acid-sensitive catchments (i.e. those with lower base cation availability), consistent with the soil mechanisms outlined above [13]. The model captures most of the regional variation in DOC trends as illustrated in boxplots of region-wise residual variation in ΔDOC (figure 4). One exception is Newfoundland, where high DOC values in the early 1990s are not adequately captured in the model (figure S3).

**Figure 5.**
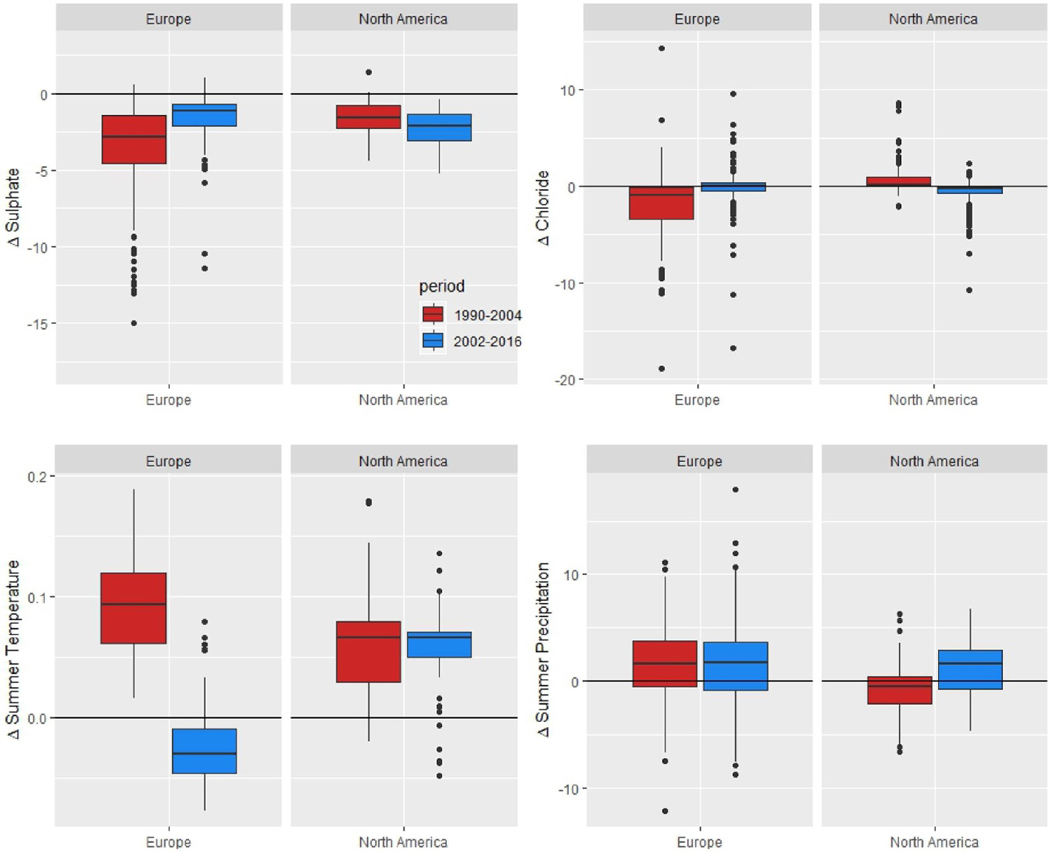
Box and whisker plots of key potential drivers of DOC change (slopes in *μ*eq l^−1^ yr^−1^ for sulfate and chloride; slopes in ° C yr^−1^ and mm yr^−1^ for temperature and precipitation, respectively), organized by continent and calculated for each of two time periods (1990–2004 and 2002–2016).

Two climate variables representing change in summer precipitation and temperature (Δsummer_P and Δsummer_T) exert small but significant, and opposing, effects, once the chemical drivers have been accounted for. Trends in both variables are predominantly upward (table 1 and figure 5), although more consistently significant for temperature than for precipitation. Increasing Δsummer_P is positively associated with ΔDOC, whereas the opposite is true for increasing Δsummer_T.

### 3.3. Regional contrasts in deceleration and acceleration of DOC trends

**Figure 6.**
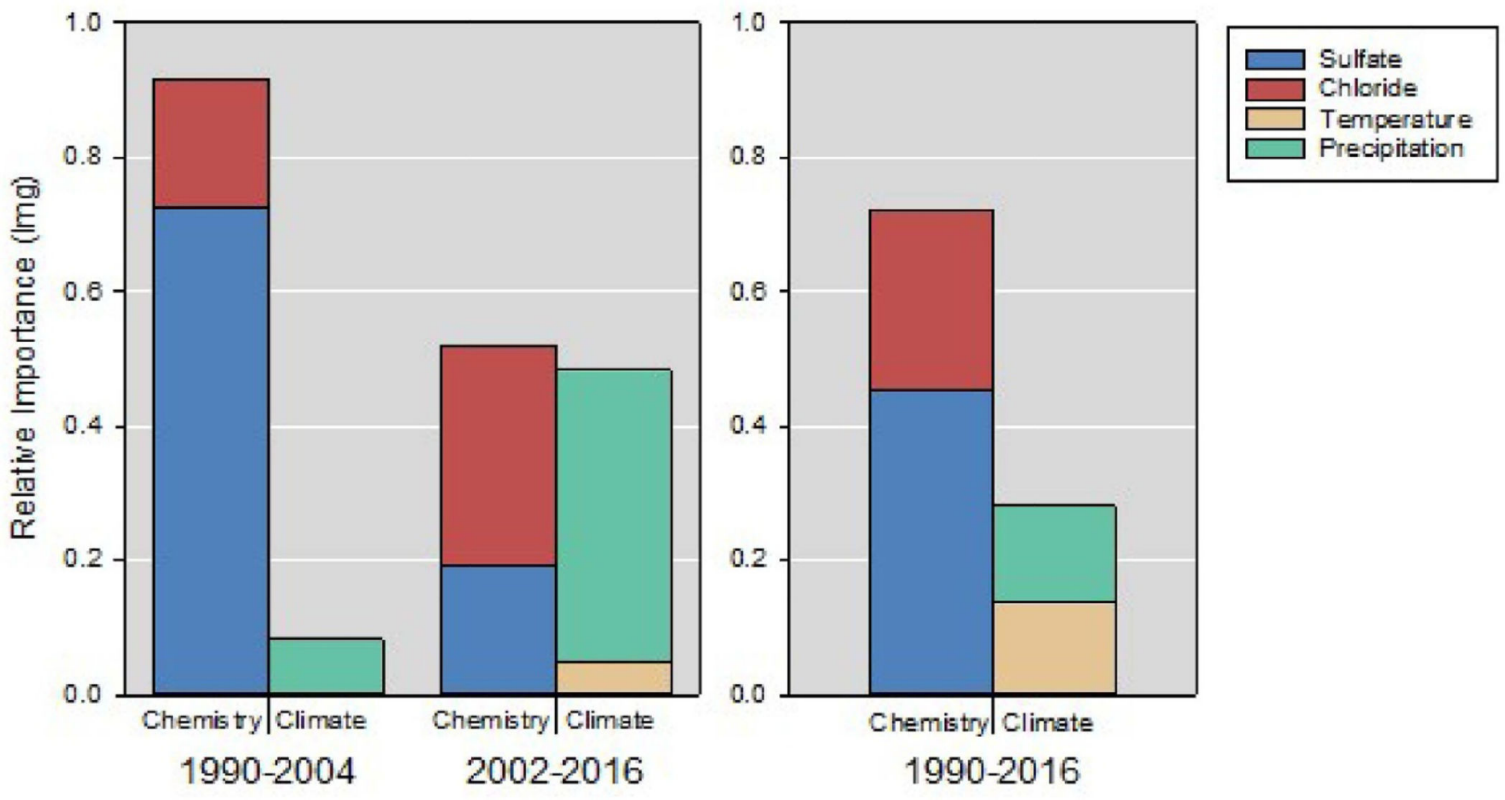
Results of relative importance analysis of candidate predictors (dynamic variables only) for DOC trends in Europe and North America for early (1990–2004), late (2002–2016) and entire (1990–2016) time periods. Relative importance expressed in ‘lmg’ which is a metric that averages relative improvements in R^2^ upon addition of predictors to multiple regression models of various sizes, in terms ofthe total variance explained.

We tested for evidence of an increased influence of climate on DOC trends, using a variance decomposition method to estimate the relative importance of all candidate predictors in multiple regression models for each period (section 2 and table 1). As we were primarily interested in the influence of dynamic predictors, we forced the analysis to include median DOC as a first step, and then calculated each driver’s contribution to the remaining variance (figure 6). For the entire data period, the relative importance analysis reinforces the conclusions from best subsets multiple regression (table 2) that the influence of the chemical drivers exceeds that of temperature and precipitation. However, the proportion of the variance explained by climatic versus chemical drivers changes. In the early period, circa 90% of the explained variance in ΔDOC by these two drivers was attributable to ΔSO_4_ and to a lesser extent ΔCl, implying that deposition reductions had driven DOC increases overwhelmingly. In the late period, however, the sum effect of trends in climate variables (predominantly precipitation) accounts for 48% of this variance, i.e. very similar to the sum effect of trends in chemical variables (52%). This change in the relative importance of climate variables is striking and suggests that these ecosystems may be moving towards a situation where climate drivers will dominate spatial variation in size and direction of DOC trends.

The accelerating and decelerating rates of browning in North America and Europe, respectively, are highly consistent with differences between regions in the temporal behavior of the dominant drivers (table 1 and figure 5). Most importantly, SO_4_ declines have slowed in Europe, but have accelerated (i.e. become more negative) in North America. Additionally, the substantial reductions in Cl concentrations in Europe in the early period did not continue in the late period, while changes in Cl concentrations reversed from slightly positive to negative in North America reflecting both the variable nature of sea-salt deposition and changes in the importance of HCl deposition. Summers in North America shifted from a drying trend in the early period to a wetting trend in the late period, while in Europe summer rainfall increased in both periods. Summers became warmer on both continents during the early time period, but this trend continued into the late period in North America only. We conclude that the contrast in acceleration of browning in North America and deceleration in Europe is driven by differential trends in cleaner air, seasalt deposition and summer precipitation.

### 3.4. Estimated changes in land to sea DOC transport related to browning

Using long-term averages of riverine water transport from publicly available data (figure S4) and median concentrations of DOC from our dataset, we estimated how riverine DOC transport to coastal regions from large drainage basins encompassing our study sites might have changed over the course of our study (table 3). This analysis suggests that the total export of DOC from these areas has increased by 27%, i.e. from 8.0to 10.7TgCyr^−1^ between 1992 and 2016 (table 3). Grouped by continent, the increase was largest in Europe (Europe: +1.7 Tg C yr^−1^ or +35%; North America: +0.7TgCyr^−1^ or +16%) which agrees with the highest absolute DOC trends and concentrations found here.

## 4. Discussion

### 4.1. Region-wide browning continues

Our analysis demonstrates that recent browning rates in headwaters of many European and North American landscapes are comparable to those reported earlier [1], i.e. at rates between 0% and 2% annually. We found no differences in relative DOC trends in the early and late period, and thus no change in browning rates. Most sites present in dataset from Monteith *et al* [1] were also included here, with the exception of Finland (number of sites declined from 157 to 26) and Ontario (number of sites declined from 32 to 15). Despite considerable differences in vegetation cover, sampling frequency, exposure to air pollution and dominating climate, simple linear models with a limited number of variables could explain two thirds of the observed variation in DOC trends. The relationships we find in this study are consistent with those reported in 2007 [1] with regard to the importance of chemical controls on DOC. However, we detected a stronger effect of climate for the 1990–2004 period, possibly because of the greater spatial resolution of available modeled climate data that was not available in the mid-2000s (i.e. 0.5 × 0.5 vs 5 × 5 degree grids). The larger impact of climate in the 2002–2016 period strongly suggests that climatic factors have become more important for browning, an observation supported elsewhere [4].

While climate change effects on biogeochemical processes controlling carbon cycling are expected to intensify, pollutant S and Cl levels in atmospheric deposition are now so low in many areas in Europe and North America that there is limited potential for further decreases [22]. Seasalt deposition and precipitation patterns appear to be related to atmospheric circulation patterns such as the North Atlantic Oscillation [25], which are inherently complex to forecast [46], rendering predictions of storms that generate seasalt aerosols very uncertain. By contrast, scientific consensus on global warming is beyond dispute [47], with both flooding and droughts expected to become more frequent. Land-use change such as forest expansion [48] could influence DOC export in some areas, but was not found to influence DOC trends in the UK sites in our study [35]. Land use evidently cannot explain either the consistency or the spatial distribution of increases that are observed across an extensive and diverse range of sites included in our dataset, many of which are located in alpine or subarctic areas with little or no forest or have grasslands as the dominant land cover.

The positive correlations we found between precipitation and DOC may be related to the increased proportion of superficial runoff through organic-rich topsoils during periods of high precipitation [49] Contrary to expectations, we found no evidence that warming has contributed to browning. Instead, we found a small, but significant, damping of DOC increases by higher summer temperatures. Studies of spatial variation have found positive relations between temperature and lake DOC [26], while century-long DOC-reconstructions from lake sediments suggest that higher precipitation and temperature both promote DOC, in addition to reduced acid deposition and land use [50]. The latter study [50] highlights that impacts of such controls are overlapping and their relative importance is difficult to ascertain. Studies of seasonal variation in DOC often show increased DOC during summer and fall, interpreted as a consequence of the temperature dependency of DOC production [26]. However, warming also promotes the mineralization of organic matter to CO_2_, and the effect of summer warming could be to switch the balance from DOC production (i.e. partial decomposition of organic matter) towards CO_2_ production [3] (i.e. complete decomposition) [51]. In an observational study covering spatial and temporal variation like the one we report here, we assume that drivers exert the same influence in space and in time, i.e. that spatial differences in acidic deposition produce similar differences in DOC behavior that temporal trends in acidic deposition would. With substantial internal correlation between the climatic and chemical drivers, we cannot know for certain whether the observed negative temperature effect is due to a spatial pattern (for example, if higher summer warming is associated with areas that also show larger changes in SO_4_) or whether it presents a shift from soil production of DOC to CO_2_.

### 4.2. Implications for the carbon balance

Drought and excess precipitation have a strong influence on net primary production [3, 52], vertical [53] and lateral [54] carbon transport and aquatic carbon cycling, the latter through the positive relationship between photochemical and microbial processing of DOM and water residence time [55]. The influence of climate on vertical and lateral transport of DOC has implications for the overall carbon balance of terrestrial ecosystems. Depending on climatic factors, different proportions of the DOC mobilized from organic soils are likely to be retained in mineral soils, exported to surface waters and re-sequestered in lake and marine sediments, or mineralized in aquatic systems and either incorporated in the ocean inorganic carbon pool or returned directly to the atmosphere. In the context of drought, lower DOC transport in a warming world could be associated with higher soil CO_2_ emissions.

Land to sea transport of carbon by rivers is not included in the most recent global carbon budget [56] although its magnitude (i.e. 1.0 Pg C yr^−1^) is significant compared with the land carbon sink and is likely to have increased significantly since the pre-industrial era [57, 58]. Our estimate suggests an increase of 27% (2.3 Tg C yr^−1^) from large watersheds in Europe and North America in the course of less than three decades. The average areal export of DOC in our study was between 1.5 and 3.4 g C m^−2^ yr^−1^ which is in the range reported for tundra, taiga and boreal ecosystems [58–60]. Inferring lateral DOC export from annual values of DOC concentrations and freshwater runoff results ignores the often disproportional contribution of snowmelt and precipitation events to DOC export [61], suggesting that our estimate of lateral C export could be too low. However, DOC retention and loss during transport from headwaters to coastal waters by aquatic processing can also be significant [55], suggesting a possible overestimation of our coastal DOC input value. Our assessment also omits any additional DOC sources that may be present in the lower reaches of river systems, for example those associated with agricultural, urban and industrial inputs and does not take interannual variations in DOC and discharge into account. Improved estimations of headwater export and subsequent aquatic processing would be valuable for constraining the role of aquatic ecosystems in the global carbon cycle, in the context where climate is becoming a more important control of DOC trends.

## 5. Conclusion

We conclude that DOC export, while still dependent on rates of acidic deposition, is becoming increasingly sensitive to changes in climate. We expect that further reductions in acidic deposition, either alone or in combination with increases in precipitation, will lead to significantly higher export of DOC from soils to surface waters. Because DOC export is intrinsically linked to the C balance of terrestrial systems, and is a major component of the land-ocean C flux, it is critical that we understand the complex interactions of changing climate, reduced atmospheric deposition and soil processes. Rates of acid deposition are beginning to approach background values in some parts of Europe and a similar situation is expected in the more industrialized regions of North America in the near future. As chemical influences on soil DOM mobility dissipate, the importance of understanding the effects of changing climate on the complex processes that determine DOC production, transport and fate cannot be understated.

## Supplementary Material

SI

## Figures and Tables

**Table 1.** Median trends, with 25%–75% quartile range in parentheses in DOC, SO_4_ + Cl (chemical drivers) and summer *T* and *P* (climatic drivers) for early (1990–2004) and late period (2002–2016). EUR, Europe. NA, North America. *p* refers to significance level of pairwise comparison between early and late period using Tukey’s *t*-test. Summer period was July–September.

	Region	Unit	1990–2016	1990–2004	2002–2016	*P*
ΔDOC	EUR + NA	mgC 1^−1^yr^−1^	0.04 (0.01–0.09)	0.03 (0.00–0.09)	0.04 (0.01–0.10)	<0.001
%ΔDOC	EUR + NA	% yr^−1^	1.1 (0.5–1.7)	0.9 (−0.1−2.3)	1.1 (0.3–1.9)	n.s.
	EUR	% yr^−1^	1.5 (0.9–2.0)	1.7 (0.5–3.1)	1.3 (0.3–2.1)	<0.0001
	NA	% yr^−1^	0.7 (0.2–1.2)	0.2 (−0.7−1.0)	0.9 (0.3–1.8)	<0.001
ΔSO_4_	EUR	*μ*eq l^−1^ yr^−1^	—	–2.8 (–4.7 to –1.4)	− 1.1 (−2.1 to −0.7)	<0.0001
	NA		—	–1.6 (–2.3 to –0.8)	−2.1 (−3.1 to −1.3)	<0.0001
ΔCl	EUR		—	–0.9 (–3.5 to –0.1)	0.0 (−0.5−0.3)	<0.0001
	NA		—	0.1 (0.0–1.0)	−0.2 (−0.7−0.0)	<0.0001
Δtemp_summer_	EUR	°C yr^−1^	—	0.09 (0.06–0.12)	−0.03 (−0.05 to −0.01)	<0.0001
	NA		—	0.07 (0.03–0.08)	0.07 (0.05–0.07)	n.s.
Δprec_summer_	EUR	mm yr^−1^	—	1.6 (−0.6−3.8)	1.7 (−0.9−3.7)	n.s.
	NA		—	−0.5 (−2.1−0.5)	1.6 (−0.8−2.9)	<0.0001

**Table 2.** Results of running best subsets regression models for DOC trends for 1990–2016. Main effects variables are ordered by their effect size (largest first). Median CaMg is the sum of Ca and Mg (in equivalents).

Variables	Coefficient	*t*-ratio	*p*
Intercept	1.68 × 10^−4^	0.0	>0.5
	Main effects		
Median DOC	1.08 × 10^−2^	17.7	<.0001
ΔSO4	−1.59 × 10^−2^	−8.7	<.0001
Median CaMg	−1.27 × 10^−4^	−4.0	<.0001
Δtemp_summer_	−5.31 × 10^−1^	−3.9	<0.001
ΔCl	−1.G5 × 10^−2^	−3.4	<0.001
Δprec_summer_	5.00 × 10^−3^	3.4	<0.001
	Interaction		
Median CaMg × ΔCl	5.06 × 10^−5^	3.8	<0.001

Goodness of fit: *r*^2^ 0.67 (adjusted *r*^2^ 0.66); AIC —1385.3; BIC —1348.8.

**Table 3.** Estimated change in riverine DOC export to coastal areas for six main drainage basins in Europe and North America, for 1992–2016.

	Area	Runoff^a^	Median DOC^b^	ΔDOC^c^	DOC flux	DOC flux	DOC flux	DOC flux

					1992	2016	2016	1992–2016
Drainage area	10^6^ km^2^	mm yr^−1^	mg C l^*−*1^(*N* sites)	% yr^*−*1^	g C m^*−*2^ yr^*−*1^	g C m^*−*2^ yr^*−*1^	Tg C yr^*−*1^	% change
Norwegian Sea	0.209	697	1.5 (16)	0.9	1.1	1.3	0.3	22
North Sea	1.001	420	3.3 (138)	1.8	1.4	2.0	2.0	43
Baltic Sea	1.660	277	6.7 (72)	1.3	1.9	2.5	4.1	32
Scotian Shelf	0.197	397	6.0 (51)	0.8	2.4	2.9	0.6	20
North East Shelf	0.538	485	3.7 (75)	0.9	1.8	2.1	1.2	21
Gulf of St Lawrence	1.349	410	4.2 (51)	0.6	1.7	1.9	2.6	14
In Europe	2.870	—	—	—	1.6	2.2	6.4	35
In North America	2.084	—	—	—	1.8	2.1	4.4	16
Total	4.954	—	—	—	1.7	2.2	10.7	27

a Area and long-term statistics (annual runoff for 1990–2016) of GRDC timeseries data/online provided by the Global Runoff Data Centre of WMO. Koblenz: Federal Institute of Hydrology (BfG), (date of retrieval: 04 August 2021).

b Median DOC in 1992 (based on 1990–1994 period) for each drainage basin. Number of sites (*N*) in each drainage basin in parentheses.

c Median annual relative change in DOC per drainage basin for period 1990–2016.

## Data Availability

Documentation of all data processing required to create the initial trends dataset and the datasets will be made available. The data that support the findings of this study are openly available at the following URL/DOI: https://github.com/JamesSample/icpw/tree/master/trends_paper_datasets.
