# Supplementary material for: Cleaner air reveals growing influence of climate on dissolved organic carbon trends in northern headwaters
Source: Environ Res Lett. Author manuscript; Available in PMC 2022 Sep 21. (PMC9306449; doi:10.1088/1748-9326/ac2526)
Supplement: SI [file NIHMS1785715-supplement-SI.docx]

**Supplementary information**

Table SI-1: Chemical, Climate and Interaction terms entered as candidate predictors in regression analyses

| Chemical Variables | Climate Variables | Interactions |
| --- | --- | --- |
| Median DOC concentration (median DOC) | Trend in Annual Precipitation (Δ Precip_annual_) | ΔSO_4_: median CaMg |
| Trend in SO_4_^2-^ (ΔSO_4_) | Trend in Annual Temperature (Δ Temp_annual_) | Δ Cl: median CaMg |
| Trend in Cl^-^ (ΔCl) | Trend in Summer Precipitation (Δ Precip_summer_) | ΔTemp_annual_: median DOC |
| Trend in NO_3_^-^ (ΔNO_3_) | Trend in Summer Temperature (ΔTemp_summer_) | ΔPrecip_annual_: median DOC |
| Median Ca^2+^ + Mg^2+^ concentration (median CaMg) |  | ΔTemp_summer_: median DOC |
|  |  | ΔPrecip_summer_: median DOC |


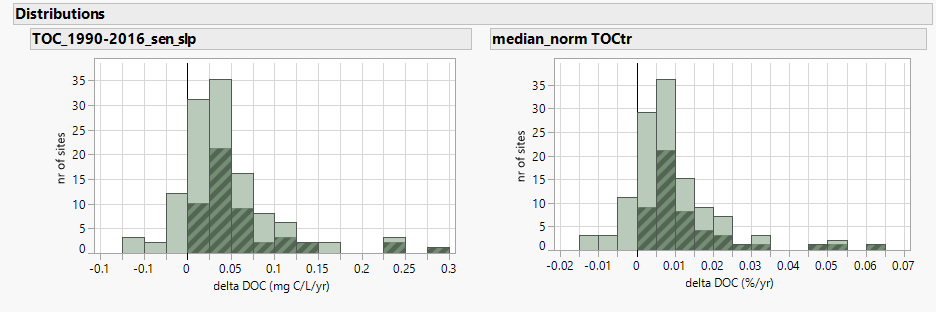

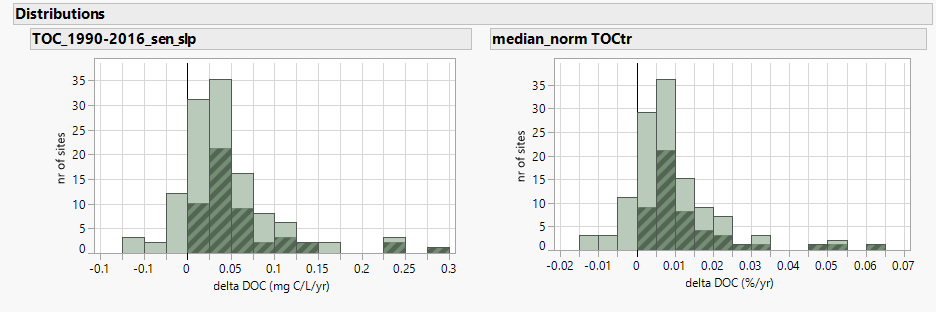


Figure SI-1. Histogram of absolute (top panel) and relative (bottom panel) trends distribution in DOC for the period 1990-2016 (slopes in mg L^-1^ yr^-1^). Light-green bars show the distribution of all slopes. Marked bars indicate slopes that are significant at p < 0.05.

Figure SI-2. Relationship between ΔDOC and median DOC concentrations for period 1990-2016, for ΔDOC≥0 (positive trends), and ΔDOC<0 (negative trends). Slopes of both regression lines are significant (p<0.0001)


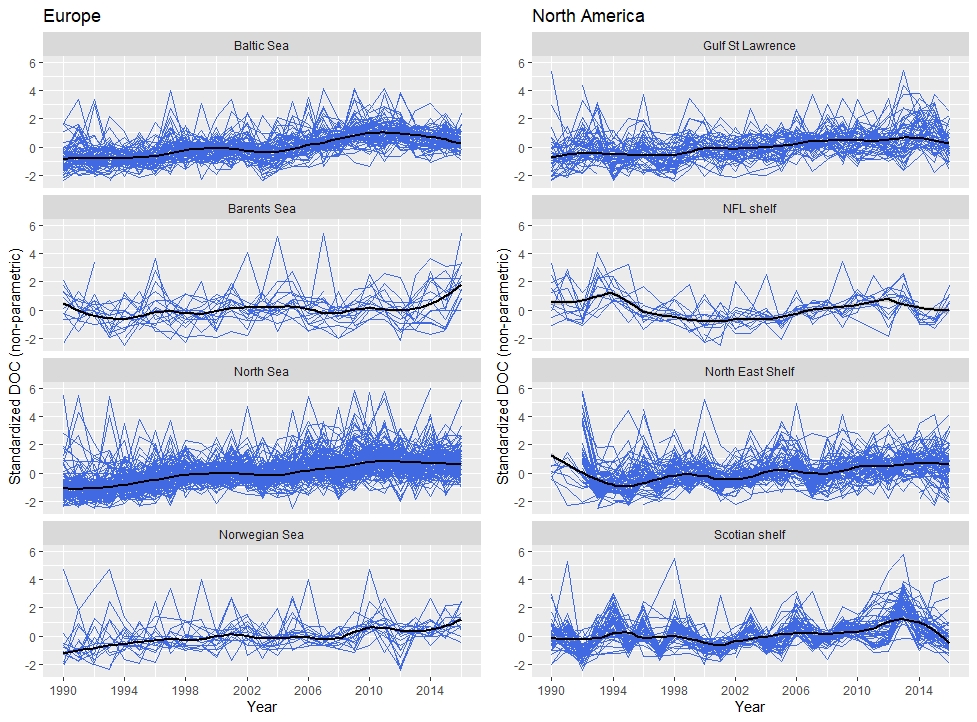


Figure SI-3. Trajectories for standardized DOC concentrations at all sites, organized by watershed (source: Global Runoff Data Centre). Each line represents an individual site. Bold blue line is Loess smoothed trajectory of the median of all sites in each watershed. NFL shelf, Newfoundland Shelf.

Figure SI-4. Annual runoff for North American (top panel) and European (bottom panel) watersheds in km^3^ yr^-1^ for the years 1990 to 2016. Linear regression lines are given (slopes not significantly different from 0). Source: GRDC Timeseries Data / Online provided by the Global Runoff Data Centre of WMO; Koblenz, Federal Institute of Hydrology (BfG). Date of retrieval: 2021-08-04.
